# *Hericium erinaceus* Improves Recognition Memory and Induces Hippocampal and Cerebellar Neurogenesis in Frail Mice during Aging

**DOI:** 10.3390/nu11040715

**Published:** 2019-03-27

**Authors:** Daniela Ratto, Federica Corana, Barbara Mannucci, Erica Cecilia Priori, Filippo Cobelli, Elisa Roda, Beatrice Ferrari, Alessandra Occhinegro, Carmine Di Iorio, Fabrizio De Luca, Valentina Cesaroni, Carolina Girometta, Maria Grazia Bottone, Elena Savino, Hirokazu Kawagishi, Paola Rossi

**Affiliations:** 1 Department of Biology and Biotechnology “L. Spallanzani”, University of Pavia, 27100 Pavia, Italy; daniela.ratto01@universitadipavia.it (D.R.); ericacecilia.priori01@universitadipavia.it (E.C.P.); filippo.cobelli01@universitadipavia.it (F.C.); elisa.roda@unipv.it (E.R.); beatrice.ferrari01@universitadipavia.it (B.F.); alessandra.occhinegro01@universitadipavia.it (A.O.); carmine.diiorio01@universitadipavia.it (C.D.I.); fabrizio.deluca01@universitadipavia.it (F.D.L.); mariagrazia.bottone@unipv.it (M.G.B.); 2 Centro Grandi Strumenti, University of Pavia, 27100 Pavia, Italy; federica.corana@unipv.it (F.C.); barbara.mannucci@unipv.it (B.M.); 3 Laboratory of Clinical & Experimental Toxicology, Pavia Poison Centre, National Toxicology Information Centre, Toxicology Unit, ICS Maugeri SpA, IRCCS Pavia, 27100 Pavia, Italy; 4 Department of Earth and Environmental Sciences, University of Pavia, 27100 Pavia, Italy; valentina.cesaroni01@universitadipavia.it (V.C.); carolina.girometta01@universitadipavia.it (C.G.); elena.savino@unipv.it (E.S.); 5 Research Institute of Green Science and Technology, Shizuoka University, Shizuoka 422-8529, Japan; kawagishi.hirokazu@shizuoka.ac.jp

**Keywords:** aging, phenotypic frailty, cognitive decline, *Hericium erinaceus*, erinacines, hericenones, medicinal mushrooms supplementation, neurogenesis

## Abstract

Frailty is a geriatric syndrome associated with both locomotor and cognitive decline, implicated in both poor quality of life and negative health outcomes. One central question surrounding frailty is whether phenotypic frailty is associated with the cognitive impairment during aging. Using spontaneous behavioral tests and by studying the dynamic change during aging, we demonstrated that the two form of vulnerability, locomotor and recognition memory decline, develop in parallel and therefore, integration of the motoric and cognitive evaluations are imperative. We developed an integrated frailty index based on both phenotypic and recognition memory performances. *Hericium erinaceus* (*H. erinaceus*) is a medicinal mushroom that improves recognition memory in mice. By using HPLC-UV-ESI/MS analyses we obtained standardized amounts of erinacine A and hericenones C and D in *H. erinaceus* extracts, that were tested in our animal model of physiological aging. Two-month oral supplementation with *H. erinaceus* reversed the age-decline of recognition memory. Proliferating cell nuclear antigen (PCNA) and doublecortin (DCX) immunohistochemistry in the hippocampus and cerebellum in treated mice supported a positive effect of an *H. erinaceus* on neurogenesis in frail mice.

## 1. Introduction

Recent reports on the European population suggest that by 2060, 30% of Europeans will be over the age of 65. Frailty is a geriatric syndrome associated with poor quality of life and negative health outcomes, such as acute illness, falls, hospitalization, disability, dependency, and mortality, adjusted for comorbidities [1,2,3], in the absence of recognized disabilities or organ-specific diseases. Health declines in frailty are accelerated and accompanied by the failure of homeostatic mechanisms [4,5].

Fried defined phenotypic frailty as an aging-associated phenotype expressing at least three of the following symptoms: weakness, weight loss, slow walking speed, fatigue, and a low level of physical activity [1]. Most older people gradually become frail and oscillations between non-frail, pre-frail, and frail are not uncommon [6]. Cognitive impairment is a decline of cognitive functions such as remembering, reasoning, and planning, ranging from mild forms of forgetfulness to severe dementia. Cognition-impaired frailty in human studies was associated with global cognition and perceptual speed, but not with episodic memory [7]. Quality of life in the elderly is particularly affected by impairments in the functioning of the memory system [8]. In order to evaluate the inclusion of cognitive performances in frailty clinical diagnosis [9], it is necessary to first determine whether phenotypic frailty is associated with cognitive impairment [10].

Several epidemiological studies have reported that higher levels of phenotypic frailty increases the risk of cognitive impairment and dementia [11,12,13], and that higher levels of cognitive impairment or dementia increase the risk of phenotypic frailty [14,15,16]. This suggests that frailty may be an early indicator for subsequent cognitive decline. Understanding the mechanisms by which phenotypic frailty is linked to cognitive impairment has implications for the management of those susceptible for both phenotypic frailty and cognitive impairment.

*Hericium erinaceus* (*H. erinaceus*) is found in Europe, Asia, North America, Oceania, and generally throughout the north temperate latitudes. In Italy, it is considered quite rare; it occurs along the Apennines mountain chain, near Sicily and Sardinia, while in the North only few sporadic sightings have been reported.

*H. erinaceus* is an edible mushroom widely used as herbal medicine, in all areas mentioned above and in a few East Asian countries. Since 1990, studies on *H. erinaceus* secondary metabolites reported several (about 70) structurally related terpenoids, such as erinacines, hericenones, hericerins, hericenes, hericenols, and erinacerins [17,18,19].

All of the above-mentioned molecules, except erinacines, share a geranyl side chain bonded to a resorcinol framework; that is, they are aromatic compounds containing the 6-alkyl-2,4-dihydroxybenzoic acid unit also known as β-resorcylate [20]. Erinacines are classified as cyathane-type diterpenoids, including 20 members of 24 different diterpenoids in *H. erinaceus* [18]. The standardization of dietary supplements from medicinal mushrooms is still in its early stages, because proper standards and protocols are lost and cannot guarantee product quality [21,22].

Existing data have suggested that there is a neuroprotective effect of dietary supplementation with *H. erinaceus* in mice subjected to middle cerebral artery occlusion [23]. Furthermore, *H. erinaceus* provided a partial recovery of intellectual function of patients with a mild cognitive impairment or against other forms of neurodegenerative diseases, including dementia and Alzheimer’s [24,25,26]. It has been shown that erinacines A–I and hericenones C–H are responsible for the neuroprotective effects of stimulating Nerve Growth Factor (NGF) [27,28] and of brain derived neurotrophic factor (BDNF) synthesis in vitro [29,30]. A possible role of polysaccharides in neuroprotection has been suggested as well [25,27,31]. Additionally, the effects of *H. erinaceus* on recognition memory and on hippocampal mossy fiber-CA3 neurotransmission in wild-type middle-aged mice was recently published [32,33]. Among the neurogenic zones, hippocampus is the most interesting area in the adult brain, because it is involved in higher cognitive function, such as memory processes and certain affective behaviors. In particular adult and persistent hippocampal neurogenesis generates new excitatory neurons in the dentate gyrus and contributes in a significant way to plasticity across the life span [34].

In the current report, we have created a frailty index for locomotor and recognition memory performance and we examined the relationship between them in aging, wild-type mice. Furthermore, we observed the effect of an *H. erinaceus* supplement (He1) containing a known amount of Erinacine A, Hericenone C, and Hericenone D on frailty. Moreover, we assessed the He1 effect on hippocampal and cerebellum neurogenesis in frailty animals, by investigating specific protein markers representative of cell proliferation activity and newborn neurons occurrence.

## 2. Materials and Methods

### 2.1. Animals

Fifteen wild-type male mice (strain C57BL-6J), starting at 11 months old, were maintained in single cages in the Animal Care Facility at University of Pavia on a 12-h light/dark cycle. Water and food were provided ad libitum. All experiments were carried out in accordance with the guidelines laid out by the institution’s animal welfare committee, the Ethics Committee of Pavia University (Ministry of Health, License number 774/2016-PR).

**Figure 1:**
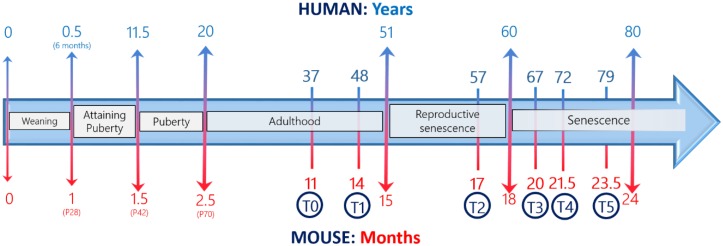
Comparative age between men and mice during their life span and the chosen experimental times (modified by Dutta and Sengupta, 2016).

In vivo experiments were performed at six different experimental times (Figure 1), between 11 and 23.5 months old.

Seven out of fifteen mice, starting from 21.5 months old, received for two months a drink made by a mixture of He1 mycelium and sporophore as ethanol extracts solubilized in water, in such a way that every mouse received 1 mg of supplement per day. This amount was chosen to mimic the oral supplementation in humans (about 1 g/day). Daily consumption of water and supplements was monitored for each mouse.

At each experimental time, mice were weighed; no statistically significant change was recorded either during aging or between the He1 and control groups.

### 2.2. Apparatus and Procedures

We performed a spontaneous behavioral test to study locomotor activity and recognition memory in mice. For all experiments, researchers were blinded to the group assignment (control and He1). Mice activity was quantified by SMART video tracking system with a selected sampling time of 40 ms/point (2 Biological Instruments, Besozzo, Varese, Italy) and Sony CCD color video camera (PAL). All mice, at different times from T0 until T5, performed two spontaneous tests, Emergence and Novel Object Recognition (NOR) tasks. Emergence and NOR tasks are used to assess recognition memory for the environment and the object, respectively.

#### 2.2.1. Emergence Test

We carried out emergence tests in accordance with procedures described by Brandalise et al., 2017 [32]. In the emergence test, we measured total distance and resting time covered in the familiar compartment as locomotor parameters, while we measured the number of exits, latency of first exit, and the time of exploration outside as cognitive indicators (Table 1).

#### 2.2.2. Novel Object Recognition Task

We carried out novel object recognition tasks in accordance with procedures described by Brandalise et al., 2017 [32], consisting of three primary phases: open arena, familiarization, and test. To assess locomotor activity, mice were observed for 15 min while freely exploring the open-field arena in the absence of objects. Locomotor parameters of mean speed, maximum speed, resting time, and the total distance covered in the arena (Table 1) were all considered. During test phase, we measured the number of approaches and the time of approaches to the familiar and the novel objects as cognitive parameters (Table 1). To evaluate the discrimination between novel and familiar objects, we calculated the Mean Novelty Discrimination Index (DI) by using the following Formula (1) [35],

DI = (*n* − *f*)/(*n* + *f*) (1)

where *n* is the average time or number of approaches to the novel object and f is the average time or number of approaches to the familiar one (Table 1). This index ranges from −1 to 1, where −1 means complete preference for the familiar object, 0 means no preference, and 1 means complete preference for the novel object.

### 2.3. The Frailty Index

A variant of Parks’s methodology [36,37] was used to calculate the Frailty Index (FI). In Parks’s procedure for creating the FI, a graded scale was calculated as follows: values that were 1 standard deviation (SD) above or below the mean reference value were given a frailty value of 0.25; values that differed by 2 SD were scored as 0.5; values that differed by 3 SD were given a value of 0.75, and values that were more than 4 SD above or below the mean received a frailty value of 1. Parameters that differed from T0 reference values by less than 1 SD received a score of 0.

Park’s procedure, as described above, was changed in order to obtain more accurate values during aging. The mean value and the standard deviation (SD) for each of the parameters were calculated at T0. The values obtained in each mouse at different times, from T0 to T5, were compared to the mean value at T0, by using the following Formula (2):

FI = (Value-Mean Value at T0)/(SD at T0)*0.25
(2)

This procedure was applied for both Locomotor FI and Cognitive FI. Finally, to obtain LAC (Locomotor And Cognitive) FI we averaged the Locomotor and Cognitive FIs.

### 2.4. H. erinaceus

The He1 (strain 1 of *H. erinaceus*) was isolated from a basidioma collected in 2013 in Siena province (Region Tuscany, Italy) from a live specimen of Quercus ilex [38]. The basidioma was aseptically cut in small portions (about 1 mm^3^) that were placed into Petri dishes with 2% malt extract agar as a culture medium (MEA, Biokar Diagnostics). Chloranphenicol at 50 ppm was added in this first step. Incubation was performed at 24 °C in complete darkness. The strain was maintained in the Italian Culture Collection of Pavia University (MicUNIPV).

#### 2.4.1. Extraction Procedures

Lyophilized mycelium and sporophores of He1 were extracted in 70% ethanol, per the procedure described by Gerbec et al. [39]. In details, one gram of dry substrate was blended with 10 mL of 70% ethanol and left in the thermostat overnight at 50 °C. Before withdrawing, the material was stirred for one hour and was centrifuged at 4000 rpm for three minutes. The supernatant was stored at −20 °C.

#### 2.4.2. HPLC-UV-ESI/MS Method

HPLC-UV-ESI/MS analyses were carried out on a Thermo Scientific LCQ FLEET system, equipped with a PAD-UV detector working at 254 nm (Thermo Scientific^®^, San Jose, CA, USA). The chromatographic separation was performed using an Ascentis Express F5 HPLC column (150 × 3.0 mm, 2.7 μm particle size Sigma Aldrich, Milan, Italy) maintained at 40 °C, with a flow rate of 0.3 mL/min and an injection volume of 20 µL. The following gradient method was utilized with water containing 0.1% formic acid (solvent A) and acetonitrile (solvent B): 0–9 min (30–50% B), 9–27 min (50–60% B), 27–54 min (60–100% B), 54–69 min (100–30% B), and 69-75 min (30% B); all solvents are from Sigma Aldrich, Milan, Italy. The HPLC system was interfaced to the ion trap mass spectrometer with an Electro Spray Ionization (ESI) ion source. The compounds were analyzed under positive (ESI+) ion conditions. The ion spray and capillary voltage were set at 5kV and 10V, respectively, in positive ion mode. The capillary temperature was 400 °C. The acquisition was performed both in Full Scan mode (mass range 200–2000 Da) and MS/MS Dependent Scan mode. The data station utilized the Xcalibur MS Software Version 2.1 (Thermo Scientific^®^, San Jose, CA, USA).

Erinacine A and hericenones C and D were used as standards [40,41]. Stock solutions (1 mg/mL) of erinacine A and hericenones C and D were prepared in 70% ethanol. Standard solutions with the final concentration range of 1–25 µg/mL for erinacine A and 20–100 µg/mL for hericenones C and D were prepared. Linear least-square regression analysis for the calibration curves showed correlation coefficients of 0.9968, 0.9945, and 0.9951, respectively, for erinacine A, hericenones C, and hericenones D with respect to the peak area, demonstrating a good linear relationship in the different ranges tested. Each concentration was analyzed in triplicate.

### 2.5. Tissue Sampling: Hippocampal and Cerebellar Specimens Preparation

Mice were anesthetized by isoflurane inhalation (Aldrich, Milwaukee, WI, USA) before decapitation.

The brain and cerebellum were immediately excised as previously described [42], washed in 0.9% NaCl, and fixed by immersion for 48 h at room temperature in Carnoy’s solution (6 absolute ethanol/3 chloroform/1 acetic acid). The tissues were then kept in absolute ethanol for one hour, followed by acetone for 50 min, and finally embedded in Paraplast X-TRA (Sigma Aldrich, Milan, Italy). Eight micron-thick sections, collected on silane-coated slides, of brain and cerebellar vermis were cut in the sagittal plane.

### 2.6. Immunohistochemistry: Fluorescence Microscopy Assessment and Quantification of Cell Proliferation and Neurogenesis

To avoid possible staining differences due to small changes in the procedure, the immunoreactions were carried out simultaneously on slides from controls and treated animals. Paraffin-embedded sections were deparaffinized in xylene, rehydrated through a series of graded alcohol treatments and rinsed in phosphate-buffered saline (PBS, Sigma).

PCNA (PC10), a 37 kDa molecular weight protein also known as cyclin, was employed as marker of cell proliferation. In particular, in cells fixed with organic solvents, the PCNA was demonstrated to be strongly associated in the nuclear regions where DNA synthesis is occurring [43]. DCX is considered a marker for neuronal precursors and migrating neuroblasts during neurogenesis recovery [44]. The presence and distribution of PCNA and DCX was assessed using commercial antibodies on murine specimens, focusing on the hippocampus and cerebellum. Brain and cerebellar sections of control and He1 mice were incubated overnight at room temperature with the primary antibody: (i) mouse monoclonal antibody against PCNA (1:600, Abcam, Cambridge, MA, USA), and (ii) goat polyclonal antibody against DCX (1:100, Santa Cruz Biotechnology, Santa Cruz, CA, USA). After washing in phosphate buffer saline (PBS), sections were incubated for one hour with the secondary antibody: (i) Alexa Fluor 488-conjugated anti-mouse (1:100, Molecular Probes, Space, Milano, Italy) and (ii) Alexa Fluor 594-conjugated anti-goat (1:100, Molecular Probes, Space, Milano, Italy),in a dark, moist chamber. Then the nuclei were counterstained for 10 min with 0.1 µg/mL Hoechst 33258 (Sigma Aldrich, Milan, Italy). After PBS washing, coverslips were mounted in a drop of Mowiol (Calbiochem, San Diego, CA, USA).

Sections were observed by fluorescence microscopy with an Olympus BX51 equipped with a 100-W mercury lamp used under the following conditions: 330–385 nm excitation filter (excf), 400 nm dichroic mirror (dm), and 420 nm barrier filter (bf) for Hoechst 33258; 450–480 nm excf, 500 nm dm, and 515 nm bf for the fluorescence of Alexa 488; 540 nm excf, 580 nm dm, and 620 nm bf for Alexa 594. Images were recorded with an Olympus MagnaFire cam and processed with the Olympus Cell F software.

Immunofluorescence quantification was performed by calculating the percentage of PCNA or DCX immunocytochemically positive nuclei or cytoplasm of nervous cells (from the hippocampus and cerebellum) of a total number (about 300) for each animal and experimental condition, in a minimum of 10 randomly selected high-power microscopic fields.

### 2.7. Statistics

Data are reported as mean ± standard error of the mean (SEM). We performed Bartlett and Shapiro Wilk Tests to establish and confirm the normality of parameters. To verify statistically significant differences, we used a one-way Anova for repeated measures of the aging of mice and a two-way Anova for the effect of *H. erinaceus* supplementation. The statistical analysis for immunofluorescence was carried out using an Unpaired Student’s t-test. The differences are considered statistically significant for *p* < 0.05 (*), *p* < 0.01 (**), *p* < 0.001 (***), and *p* < 0.0001 (****). Statistical analyses were performed with GraphPad Prism 7.0 software (GraphPad Software Inc., La Jolla, CA, USA).

## 3. Results

### 3.1. Locomotor and Recognition Memory during Physiological Aging

We first investigated locomotor performance and recognition memory during physiological aging in healthy mice (*n* = 15) using different spontaneous behavioral tests. Novelty recognition memory for a new environment and for novel objects was tested by way of emergence and NOR tasks, respectively.

We carried out behavioral spontaneous tests in mice at 11 (T0), 14 (T1), 17 (T2), 20 (T3), 21.5 (T4), and 23.5 (T5) months old. For the reader to understand the practical application of these tests, a comparison of the different developmental stages between humans and mice during their life span, according to Dutta and Sengupta is outlined in Figure 1 [45]. To monitor the physiological aging in mice, we choose six experimental times: T0 and T1 corresponding to adulthood phase, T2 to reproductive senescence, and T3, T4, and T5 to senescence phase.

**Figure 2:**
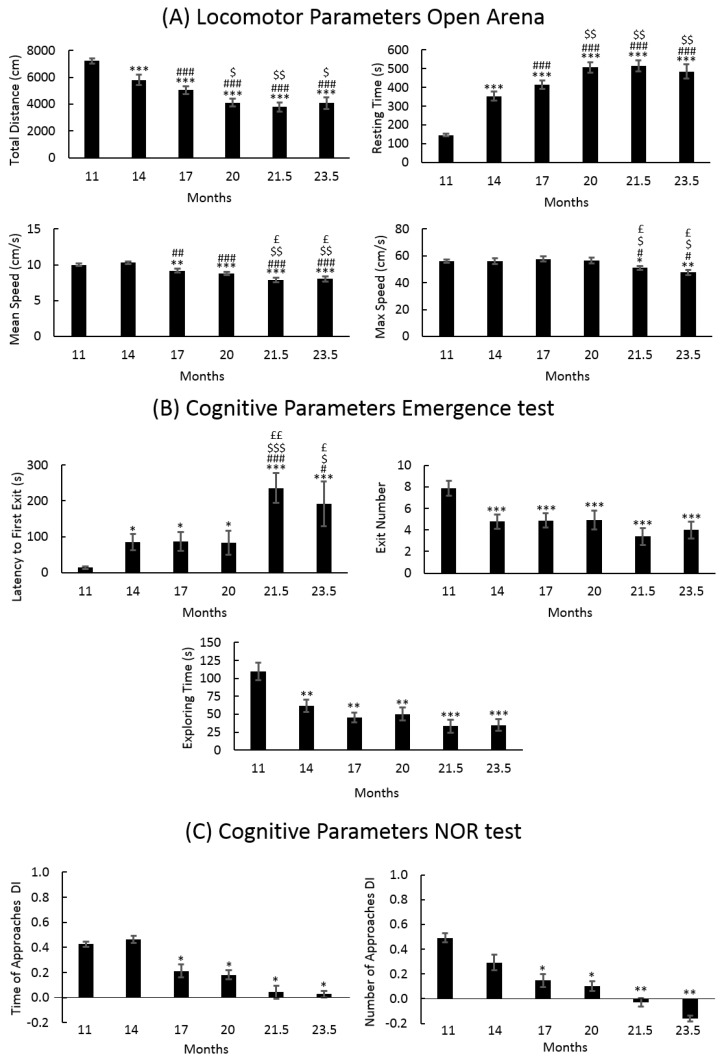
Locomotor and cognitive parameters during aging. (**A**) Locomotor parameters: total distance, resting time, mean speed, and max speed measured in open arena during aging. (**B**) cognitive parameters: latency to first exit, exit number, and exploring time measured in emergence, and (**C**) cognitive parameters: discrimination index (DI) of the time of approaches and of the number of approaches measured in NOR test. Statistical results were performed by Anova for repeated measures: * vs. T0, # vs. T1, $ vs. T2, and £ vs. T3. For all symbols reported *p* < 0.05 (*, #, $, £), *p* < 0.01 (**, ##, $$, ££), *p* < 0.001 (***, ###, $$$).

Figure 2A shows the locomotor parameters measured in the open arena during aging. Total distance and resting time decreased from T0 to T1, stabilizing at T3. Mean speed in the open arena changed at T2 and then worsened with the aging. Maximum speed worsened later, in senescence phases T4 and T5.

Figure 2B,C show the cognitive parameters measured in emergence and in NOR, respectively. In the emergence test latency to the first exit, the exit number and the exploring time worsened from T0 to T1 and then remained relatively stable, whereas the latency to the first exit worsening again in the senescence phase. In the NOR test, the time of approach and the number of approaches decreased in T2 and even more in senescence phase.

#### 3.1.1. Locomotor Frailty Index

**Figure 3:**
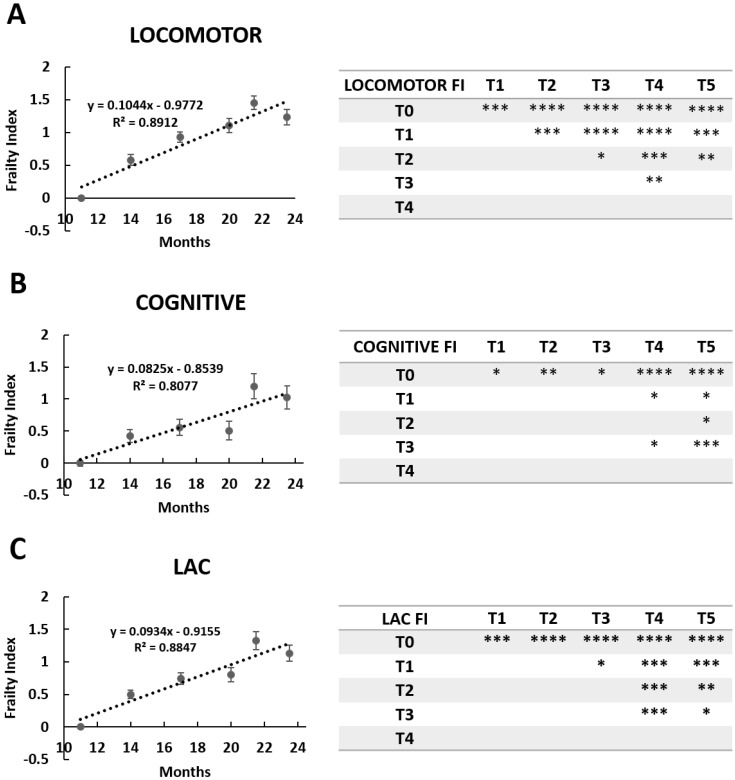
Locomotor, cognitive, and LAC (locomotor and cognitive) decline during physiological aging in mice. Locomotor (panel (**A**)), cognitive (panel (**B**)), and LAC (panel (**C**)) Frailty Index during physiological aging in mice. Linear regressions of experimental points and statistical results were reported. *p* < 0.05 (*), *p* < 0.01 (**), *p* < 0.001 (***), *p* < 0.0001 (****).

We calculated the Locomotor Frailty Index (FI) for each of the parameters reported in Table 1, then averaged the values for each experimental time. Figure 3, Panel A shows the Locomotor FIs from T0 to T5 and the linear least-square regression analysis, with R^2^ = 0.8912. Results from a one-way ANOVA are reported in Figure 3. These results suggest that during physiological aging, locomotor performances decline linearly from the adulthood to the senescence stage.

The slope obtained by the linear least-square regression analysis (slope value = 0.1044) indicated that for every three months passed, locomotor activity decreased by about 35.43%, yielding a significantly different Locomotor FI value from the previous one each time.

#### 3.1.2. Cognitive Frailty Index

Novelty recognition memory was evaluated, resulting in a calculation of the Cognitive Frailty Index (FI) during aging (Figure 3B). Cognitive FI was calculated using a linear least-square regression analysis, with R^2^ = 0.8077. Results from a one-way ANOVA are reported in Figure 3.

The slope obtained by the linear least-square regression analysis (slope value = 0.0825) indicated that for every 3 months passed, recognition memory declined by about 26.04%, yielding a significantly different Cognitive FI value the previous one each time.

#### 3.1.3. Cognitive Frailty Index

In order to evaluate a global trend of both phenotypic and cognitive decline in physiological aging, we calculated the Locomotor and Cognitive FI (LAC frailty index) by averaging all frailty indices obtained prior to evaluating the locomotor and cognitive performances during aging (Figure 3C). The LAC FI significantly increased from T0 to T5. Similarly, as previously described for Locomotor FI and Cognitive FI, the LAC FI values follow a linear trend (R^2^ = 0.8847) (Figure 3C). The slope obtained by the linear least-square regression analysis (slope value = 0.0934) indicated that for every 3 months passed, the frailty of the mouse increased by about 30.7%.

### 3.2. Identification and Quantification of Erinacine A, Hericenone C, and Hericenone D

In order to identify and quantify the bioactive metabolites of interest, we analyzed the mycelium and sporophore extracts of He1 using HPLC-UV-ESI/MS. Erinacine A and hericenones C and D were identified by comparing both retention times and ESI/MS spectra with the authentic standards.

**Figure 4:**
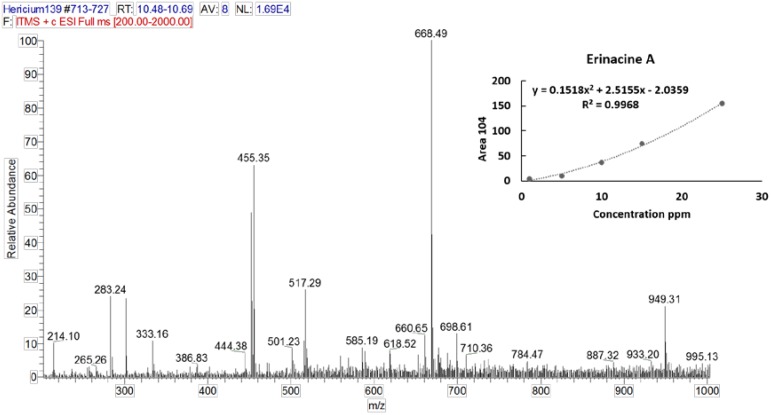
ESI/MS spectrum of Erinacine A. Panel (top, right) reports calibration curves and linear regression curve for Erinacine A.

**Figure 5:**
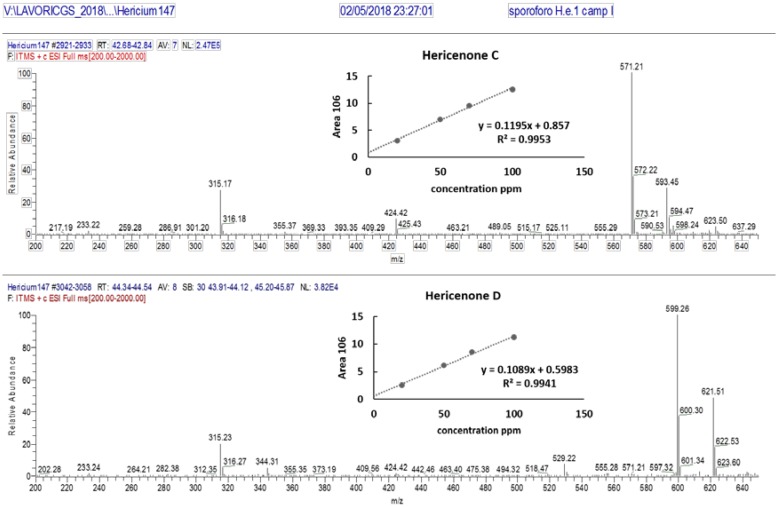
ESI/MS spectra of Hericenone C and D. Panels (top, right) report calibration curves for Hericenone C and D.

Characteristic ions of Erinacine A in the ESI/MS spectrum (Figure 4) are sodium and potassium adducts of a single molecule as well as a dimer (Table 2). Hericenone C and D spectra (Figure 5) present just [M+H]+ and [M+Na]+ ions (Table 2).

**Figure 6:**
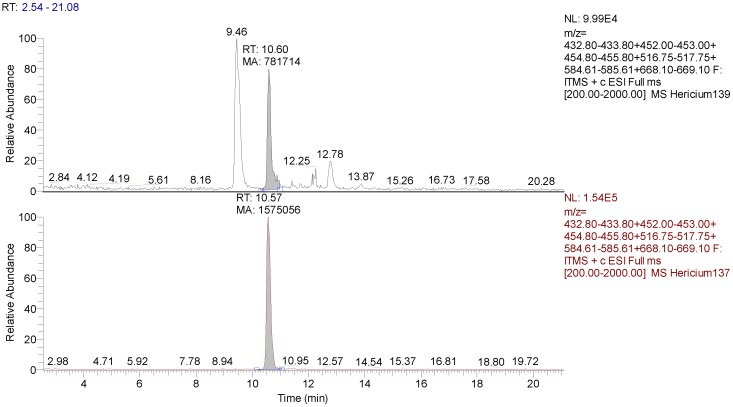
MS (Mass Spectrum) traces of He1 mycelium and Erinacine A (Rt 10,57) standard. Peak area of Erinacine A is pointed out.

Erinacine A was detected using HPLC/ESI-MS. By comparing the retention time and molecular ion or mass spectra, we confirmed the peak identification. We quantified it by comparing the peak areas with those of the standard (Figure 6). The calibration curve was constructed by injecting standard mixture solutions at five concentrations (1, 5, 10, 15, and 25 µg/mL). The linear least-square regression analysis for the calibration curve showed a correlation coefficient of r = 0.9968. The level of Erinacine A present in mycelium of He1 calculated by the calibration curve was 150 µg/g (Table 2).

**Figure 7:**
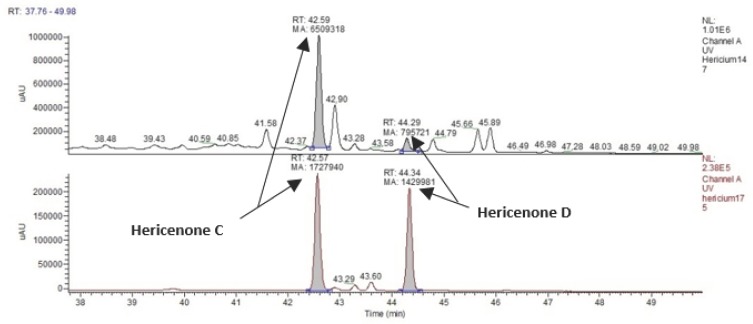
UV (Ultra Violet) traces of He1 sporophore (top) and Hericenone C (Rt 42.57) and D (Rt 44.34) standards (bottom). Peak areas corresponding to Hericenone C and D are pointed out.

The hericenones C and D chromatographic conditions produced a good resolution of adjacent peaks. UV detection provided sufficient sensitivity for each analyte, allowing proper quantification of both compounds by comparing the peak areas in the UV trace with those of the standards (Figure 7). Calibration curves were constructed by injecting the standard mixture solutions at four concentrations (20, 50, 75, 100 µg/mL.). Linear least-square regression analysis for the calibration curves showed correlation coefficients of r = 0.9945 and r = 0.9951, respectively, for hericenones C and D. The levels of hericenones C and D present in He1 basidioma, calculated by calibration curves, were 500 µg/g and less than 20 µg/g, respectively (Table 2).

### 3.3. He1 Supplementation Improved Recognition Memory Performances during Aging

We investigated cognitive and locomotor performances after oral *H. erinaceus* supplementation on frail mice. Seven mice with a T4 LAC FI score measured of more than 1.30 received a mixture of components made by He1 mycelium and basidioma for two months until T5.

**Figure 8:**
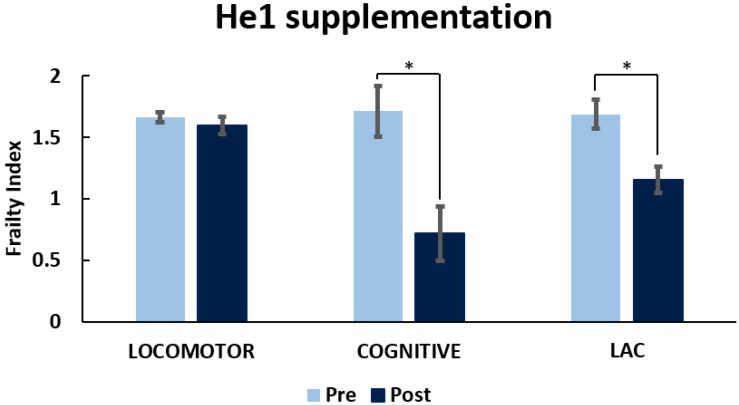
*H. erinaceus* improved recognition memory during mice aging. Value measured pre-supplementation (pre) and post-supplementation (post) on locomotor, recognition memory, and LAC (Locomotor and Cognitive) FI. *p* < 0.05 (*).

Figure 8 shows the frailty index before (T4) and after He1 supplementation (T5). He1 supplementation improved recognition memory in mice during aging, characterized by a Cognitive FI decrease from 1.71 ± 0.21 to 0.72 ± 0.22. Locomotor performances before and after He supplementation were not significant different. Considering together locomotor and memory performances by means of the LAC index, He1 regressed aged-related frailty, but this change in LAC index was completely driven by the improve in memory function.

### 3.4. He1 Supplementation Improved Hippocampal and Cerebellum Proliferation and Neurogenesis

To examine the molecular mechanism involved in the He1 effect on aging mice, we performed immunocytochemical studies on different cerebral tissues, i.e., the hippocampus and cerebellum, testing both the proliferating the cell nuclear antigen (PCNA) and doublecortin (DCX) as specific markers of active proliferation and neurogenesis, respectively. We performed immunocytochemistry on He1 frail mice and on untreated (control) animals at T5.

**Figure 9:**
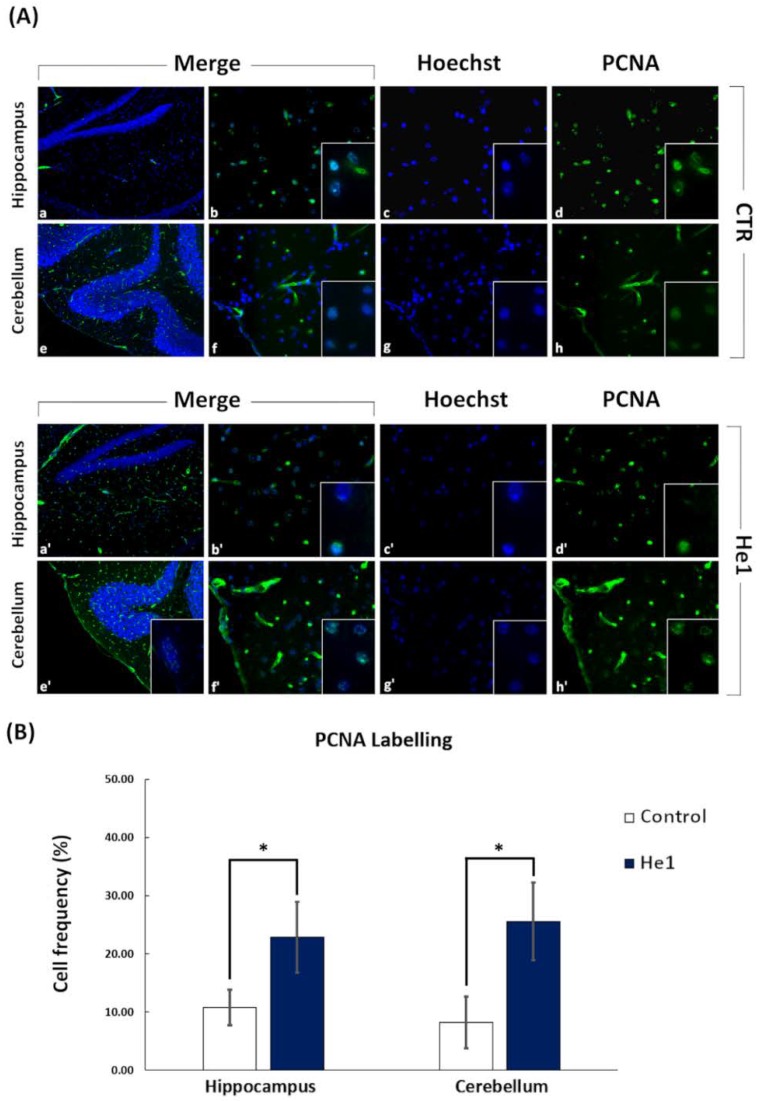
Panel (**A**) shows cell proliferating activity immunocytochemically detected by PCNA (Proliferating Cell Nuclear Antigen) labelling, observed at T5 after 2 months oral supplementation with He1 in both hippocampus and cerebellum, (a’–d’ and e’–h’, respectively), compared to control untreated mice (CTR) (a–h). Cell proliferation was significantly enhanced in He1 mice, with the labelling appearing more intense in the hippocampal DG granule cells and in CA3 pyramidal neurons (a’–d’) and in cerebellar molecular layer (e’–h’), compared to controls (a–d and e–h, respectively), predominantly localized in the DG granule cells and in CA3 pyramidal neurons, as also in the width of the cerebellar. Objective magnification: 20 x (a, e and a’, e’); 40 x (b–d, f–h and b’–d’, f’–h’); 100 x (insert in b–d, f–h, b’–d’, f’–h’). Panel (**B**) shows changes in the percentage of PCNA labelling index of hippocampal and cerebellar cells in He1 mice. *p* < 0.05 (*).

Preliminary data seem to demonstrate that cell proliferating activity achieved the highest expression in both brain areas in He1 mice compared to control animals. Specifically, the PCNA nuclear immunolabelling appeared more marked in the hippocampal dentate gyrus (DG) granule cells and in the CA3 pyramidal neurons, while the immunopositive cells (possibly granular or glial cells) predominantly localized in the width of the outer molecular layer in the cerebellar cortex (Figure 9A). Notably, clusters of PCNA-positive cells, possibly newborn granule cells, were observed suggesting the occurrence of a recovered proliferation wave (Figure 9A). Accordingly, quantitative analysis demonstrated that the PCNA labelling frequency detected in He1 treated mice (22.89% ± 6.09) reached significantly higher values compared to those measured in controls (10.80% ± 3.09, *p* < 0.05) in the hippocampus. Similarly, the PCNA labelling frequency detected in He1 mice (25.60% ± 6.66) reached significantly higher values compared to those measured in controls (8.19% ± 4.43, *p* < 0.05), in the cerebellum (Figure 9B).

**Figure 10:**
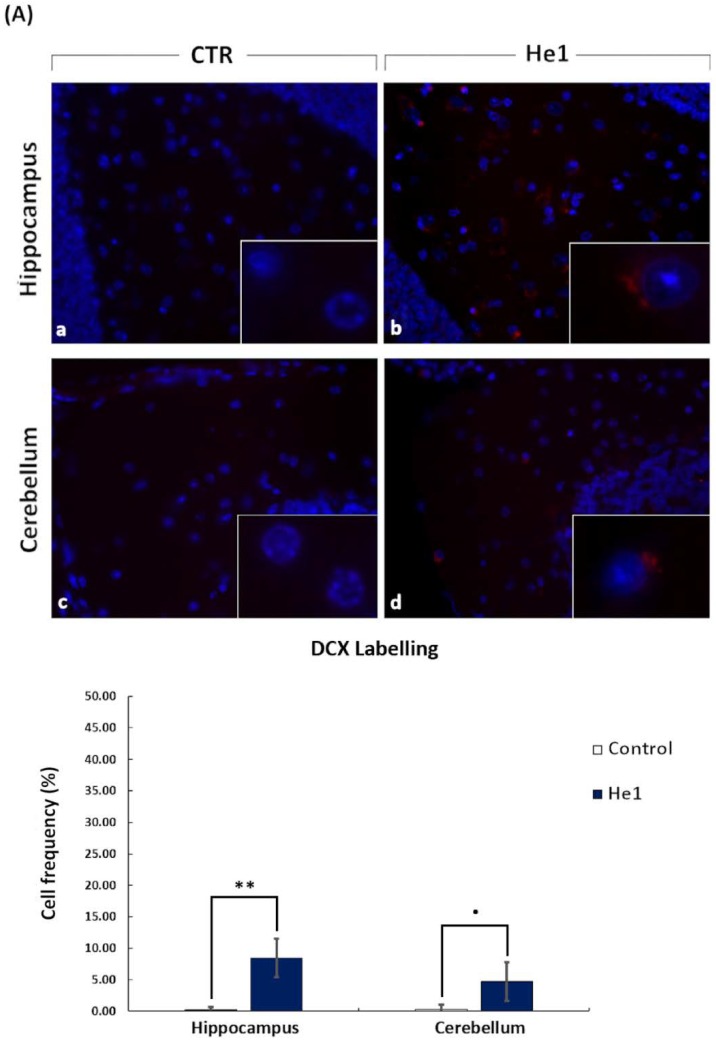
Panel (**A**) shows doublecortin (DCX) immunocytochemistry, observed at T5 after 2 months oral supplementation with He1 in hippocampus and cerebellum (b and d), compared to control mice (a and c). Objective magnification: 40 x (a–d); 100 x (inserts). Panel (**B**) shows the cell frequency percentage of DCX labelling in the hippocampal dentate gyrus and cerebellar molecular layer in control and He1 mice. *p* < 0.01 (**), *p* = 0.07 (**·**).

In He1 mice, hippocampus showed a more marked DCX labelling compared to control animals. In particular, DCX immunolabelling appeared more intense in the dentate gyrus (DG) granule cells in the hippocampus (Figure 10A, panel a and b). In the cerebellar cortex the immunopositivity was less expressed, nonetheless mainly localized in cells present in the molecular layer (Figure 10A, panel c and d). Quantitative analysis showed that the DCX labelling frequency in He1 mice achieved significantly higher values compared to those measured in control animals in hippocampus (8.45% ± 3.02 vs. 0.22% ± 0.45, respectively, with *p* < 0.05). In the cerebellum the DCX labelling frequency displayed a trend of increase in He1 mice but did not reached the threshold for the statistically significant difference compared to controls (4.68 ± 3.06 vs. 0.26% ± 0.79, respectively, with *p* = 0.073) (Figure 10B). It should be noted that the DCX labelling frequency in control animals is only about 0.2% in both hippocampus and cerebellum. As the PCNA labelling identifies DNA repair as well as duplication, and DCX positivity links to the presence of newborn neurons, the increased expression of this cytochemical marker may be the manifestation of different biological responses involving the recovery of cell proliferation and neurogenesis, potentially highlighting the occurrence of an upswing phase owed to the neurobiological effects exerted by the oral supplementation with He1.

## 4. Discussion

The aim of this study was to develop an index to monitor locomotor and cognitive frailty in aging mice in vivo and to study the effect of *H. erinaceus* extracts containing a known amount of erinacines A and hericenones C and D. The mechanism of action of He1 was evaluated by immunocytochemistry.

We developed a frailty index score (LAC FI) based both on locomotor and cognitive decline during aging. To achieve a translational approach, we chose to monitor locomotor indicators that compare well to phenotypic frailty indicators in humans, such as gait speed and the level of physical activity [1]. We therefore measured the mean and maximum speed, resting time, and the total distance covered in an open arena test.

In concordance with Fried’s phenotype model [1] and with Parks observations on activity level [36], the current paper demonstrates that the locomotor performances of mice progressively worsened during aging. The locomotor FI increased significantly during aging, with a linear progression from 11 to 23.5 months. We have developed a simplified, non-invasive method to monitor the development of frailty during mice aging, using a FI measured according to Whitehead [37].

Researchers use often the Stenberg Item Recognition Paradigm (SIRP) to measure cognitive impairment during aging in humans [46,47], during which a small group of items, called the positive set, is presented for the subject to memorize. After a delay, a single item is presented that may (familiar) or may not have (novel) been shown before and the subject is asked to answer “yes” or “no” to indicate their recognition of the item. The NOR and the emergence test in mice assess the same ability to recognize a familiar and a novel object [48] or a new environment. We measured exit numbers, the latency of the first exit and the time of exploration in the emergence test and the number and time of approaches in the NOR test [32] as cognitive parameters. We preferred to use different parameters, as suggested by Ennaceur et al., because it has been recognized that this methodology supports the validity and interpretation of the data of a behavioral experiment [49].

Locomotor and Cognitive Frailty Index scores were interpolated by a straight line. The slope of the linear regression indicates that locomotor performances decreases at a steeper rate than cognitive performances during the mouse’s life span. Therefore, the data, therefore, indicate that locomotor frailty is associated with lower performance in recognition memory. These data suggest that when mice meet frailty criteria, they should be seen as mice at risk of cognitive decline.

Standardization, efficacy, and the mechanism of action of medicinal mushroom products is a pressing problem [21]. Thanks to the comparison with standard measures, we have identified and quantified erinacines A in the mycelium, and hericenones C and D in the sporophore of He1 using the HPLC-UV-ESI/MS technique. It is worth noting that the content of 0.15 mg/g of erinacine A present in mycelium in this *Hericium* strain is comparable to that reported by Krzyczkowski et al. under the use of the most favorable combination of nutrients [50]. By monitoring the temperature and ventilation during the processing, Chen et al. subsequently obtained the highest content of erinacine A, suggesting a carbon-to-nitrogen ratio of 6 and a pH value of 4 may be important parameters in promoting the biosynthesis of erinacine A [51].

Basidioma of *H. erinaceus* contains a considerable quantity of bioactive molecules such as hericenones. The quantity of hericenones C and D in our sample of *H. erinaceus* was 0.5 mg/g and 0.02 mg/g, respectively, similar to that reported by Lee et al. in some wild-type and local varieties of *H. erinaceus* strains [52].

To be able to best generalize our results to humans, we decided to use an amount of the mycelium and basidioma extracts to mimic the supplementation used in humans. It should be noted that in vitro and in vivo effects of erinacine A and hericenones on NGF synthesis [53], on reducing amyloid burden [51], on reducing amyloid plaques, and on recovering from impairments in Morris water maze tasks [54] were obtained by using a daily millimolar concentration starting from 1-until 30 mg/Kg body weight [55]. In our experimental condition, we used 100-fold lower concentration.

Using the same experimental condition, we previously described the effect of *H. erinaceus* on improving recognition memory and the increase in spontaneous and evoked excitatory synaptic current in mossy fiber-CA3 synapses [32,33]. Similarly, Rossi et al. showed that two months of *H. erinaceus* treatment increased locomotor performances in mice [33]. The current paper confirms that He1 supplementation may increase recognition memory performance in mice during aging and may also revert the cognitive decline in frail mice.

Hippocampal neurogenesis is pivotally involved in higher cognitive function and, new excitatory dentate gyrus (DG) granule cells, generated by adult hippocampal neurogenesis, contributes significantly to neural plasticity throughout the entire life duration. Our data showed in hippocampus the recovery of cell proliferation in DG granule cells and CA3 pyramidal neurons and the presence of progenitor cells in DG granule cells. These data are in accordance with a recent in vivo study by Ryu et al. [56], supporting the notion that *H. erinaceus* extract administration promotes hippocampal neurogenesis in the adult mouse brain.

There is, currently, a dearth of literature available on the cerebellum area. An in vitro investigation showed the ability of *H. erinaceus* to promote the normal development of cerebellar cells, demonstrating a regulatory effect on the myelin genesis process [57]. Recently, Trovato and colleagues [58] demonstrated in vivo the neuroprotective action of *H. erinaceus* through the up-regulation of lipoxin A4 and modulation of stress responsive vitagene proteins. Our data supports the occurrence of a cell proliferation upswing in the cerebellum, as evidenced by the presence of several PCNA-immunopositive cells, possibly granular cells. Traditionally, the adult cerebellum is commonly considered as a “non-neurogenic” area. Nonetheless, our present results concerning cerebellar cortex demonstrated the presence of DCX-immunopositive cells localized in the molecular layer of He1-treated mice, suggesting the occurrence of newborn immature nervous cells.

In accordance with our preliminary results, recent investigations hypothesized the existence of stem cell populations within the cortex of the adult cerebellum that express stem cell markers and that can give rise to neuronal progeny when expanded in vitro and subsequently transplanted back into the murine cerebellum [59]. Therefore, further in-depth experiments, testing additional molecular markers, need to be carried out to confirm these data, initially identifying the PCNA-immunopositive proliferating cell type and then corroborating the occurrence of a “non-canonical” cerebellar neurogenic process in adult mice.

## 5. Conclusions

In conclusion, we suggest that during aging, the two form of vulnerability in locomotor and cognitive performances develop in parallel and therefore, we need to integrate motoric and cognitive evaluations. As suggested by Lauretani et al., an investigation of the “brain-muscle loop” in a simultaneous assessment of all aspects that may progressively lead to loss of independence is imperative [60]. Furthermore, *H. erinaceus* is a seemingly good candidate to regress recognition memory decline during aging, possibly through an increase in neurogenesis in the hippocampus and cerebellum. These findings rise the possibility that *H. erinaceus* extracts could be a new therapeutic strategy for preventing or treating neurodegenerative diseases such as dementia and Alzheimer’s, as suggested by other authors [24,25,26]. Future studies should investigate the mechanisms involved in this at a cellular level.

## Figures and Tables

**Table 1:** Selected parameters to measure locomotor and cognitive performances in each task. DI = discrimination index between novel/repositioned and familiar object. NOR = novel object recognition.

Test	Locomotor Parameters	Cognitive Parameters
**Emergence**	Resting Time In (s) Total Distance In (cm)	Exit Number (*n*) Latency of First Exit (s) Time of Exploration (s)
**NOR**	**(Open Arena)** Resting Time (s) Total Distance (cm) Max Speed (cm/s) Mean Speed (cm/s)	Number of Approaches: DI Time of Approaches: DI

**Table 2:** Shows the Erinacine A, Hericenones C and D molecular formula, molecular weight, chemical structure, characteristic structure, and content in 1 g of dried He1 mycelium and sporophore.

	Molecular Formula	Molecular Weight (g/moL)	Chemical Structure	Characteristic Ions (m/z)	Content (µg/g)
**Erinacine A**	C_25_H_36_O_6_	432.56	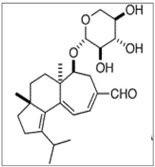	455 [M+Na]^+^ 452 [2M+K+H]^2+^ 668 [3M+K+H]^2+^ 949 [2M+HCOOH+K]^+^	150 in mycelium
**Hericenone C**	C_35_H_54_O_6_	570.81	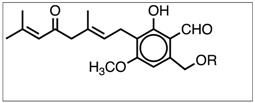 R = Palmitoyl	571 [M+H]^+^ 593 [M+Na]^+^	500 in basidioma
**Hericenone D**	C_37_H_58_O_6_	598.87	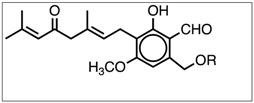 R = Stearoyl	599 [M+H]^+^ 621 [M+Na]^+^	<20 in basidioma
